# Novel Prediction of Diagnosis Effectiveness for Adaptation of the Spectral Kurtosis Technology to Varying Operating Conditions

**DOI:** 10.3390/s21206913

**Published:** 2021-10-19

**Authors:** Stuart Kolbe, Len Gelman, Andrew Ball

**Affiliations:** Department of Engineering and Technology, The University of Huddersfield, Huddersfield HD1 3DH, UK; stuart.kolbe@hud.ac.uk (S.K.); A.Ball@hud.ac.uk (A.B.)

**Keywords:** spectral kurtosis, adaptation, vibration analysis, diagnostics, online condition monitoring, machine learning

## Abstract

In this paper, two novel consistency vectors are proposed, which when combined with appropriate machine learning algorithms, can be used to adapt the Spectral Kurtosis technology for optimum gearbox damage diagnosis in varying operating conditions. Much of the existing research in the field is limited to test apparatus run in constant and carefully controlled operating conditions, and the authors have previously publicised that the Spectral Kurtosis technology requires adaptation to achieve the highest possible probabilities of correct diagnosis when a gearbox is run in non-stationary conditions of speed and load. However, the authors’ previous adaptation has been computationally heavy using a brute-force approach unsuited to online use, and therefore, created the requirement to develop these two newly proposed vectors and allow computationally lighter techniques more suited to online condition monitoring. The new vectors are demonstrated and experimentally validated on vibration data collected from a gearbox run in multiple combinations of operating conditions; for the first time, the two consistency vectors are used to predict diagnosis effectiveness, with the comparison and proof of relative gains between the traditional and novel techniques discussed. Consistency calculations are computationally light and thus, many combinations of Spectral Kurtosis technology parameters can be evaluated on a dataset in a very short time. This study shows that machine learning can predict the total probability of correct diagnosis from the consistency values and this can quickly provide pre-adaptation/prediction of optimum Spectral Kurtosis technology parameters for a dataset. The full adaptation and damage evaluation process, which is computationally heavier, can then be undertaken on a much lower number of combinations of Spectral Kurtosis resolution and threshold.

## 1. Introduction

Vibration signal analysis is a well-known and commonly used technique for condition monitoring of rotating machinery, particularly, gearboxes and bearing assemblies. In laboratory environments, these data are typically captured from test apparatus operated in constant speed and load conditions for its entire lifetime, which, whilst allowing for finely tuned algorithms and complex processing, is not necessarily applicable to industry.

McFadden is credited as having begun what are now considered the standard processing techniques of vibration data, particularly with his initial suggestion of the Time Synchronous Average (TSA) [1,2]. Badaoui et al. [3] continued this work by building complex finite element analysis models to theoretically prove the application of TSA techniques to meshing teeth and gearbox components, and compare the diagnostic technologies suggested by McFadden. Bonnardot et al. [4] proved Badaoui’s theoretical proposals on real gearboxes. They continued on to develop angular resampling in order to remove the effects of speed fluctuations on the TSA process, which was further evolved by Combet and Gelman [5] to no longer require a tachometer signal.

The Spectral Kurtosis (SK) has been observed to be sensitive to non-stationary components of a vibration signal, and can also indicate the frequencies at which these signal elements occur [6,7,8]. Coincidentally, the Wiener filter—a particularly effective de-noising filter—has a mathematical composition closely linked to the Spectral Kurtosis; so can be utilised to isolate and accentuate small non-stationary damage related transients in a vibration signal [8]. A probability of correct diagnosis can be evaluated using automated decision making algorithms which combine k nearest neighbours, cluster analysis and weighted majority scores to identify [9,10].

Damage diagnosis of rotating machinery operated in varying speed and load conditions is known to be complex with unique issues; most notably, because the vibration diagnostic features are dependent on operating conditions [11], torque changes modulate signal components [1,12,13], and, in certain situations, signal components can completely disappear while significantly changing sideband content [14]. Additional complexity can be encountered if torque changes are able to affect the gearbox speed, for which methods have been devised to compensate [15,16]. These complexities and concerns are identical regardless of the mechanism for operating condition changes as the vibration signal elements change in all cases. Changes in vibration signal components can, therefore, come from many different sources other than the presence of damage, which impedes the reliability of many conventional condition monitoring techniques [17], and, therefore, much of the ongoing research focuses on generating new and novel diagnostic indicators [11,18,19] or new signal pre-processing techniques [17], whereas, the authors’ previous work has focussed on adapting classical SK techniques to the changing operating parameters [20,21].

It has been shown, that with correct adaptation of the SK technology parameters, the total probability of correct diagnosis of a gearbox can be optimised for different combinations of speed and load, allowing continuous condition monitoring and damage detection in all operational states. Adaptation of SK methodologies was initially chosen as an investigative area because there has been a precedent of adaptive techniques in vibration signal processing, notably in gearbox state detection [22], filtering [23] and the adaptation of both wavelet and classical residual technologies [24]. Machine learning based approaches are becoming increasingly popular in the field of condition monitoring and vibration analysis as they have the potential to improve the extraction of diagnostic features and enhance the accuracy and efficiency of diagnosis [25,26].

However, adaptation of SK technologies can be computationally heavy due to the amount of processing involved. The outcomes of [20] are to conclude, that by adapting the SK resolution and SK threshold to gearbox operating conditions, the total correct diagnosis probability could be significantly increased in all operating conditions, outperforming the existing methods for selecting the main parameters of the SK technology. However, it was also stated that, while enhancing effectiveness of SK diagnosis, the proposed adaptation is computationally heavy and up to this point a ‘brute force’ approach had been used. This highlighted drawbacks to using the proposed adaptation in real world scenarios, predominantly, for on-line condition monitoring of gearboxes. So, novel, computationally light and effective methods of SK adaptation to a variable gearbox load are proposed here. It is, therefore, proposed that developing two novel vectors, that could predict and adapt the SK technology, would improve computational times, improve effectiveness of the technology and increase the relevance to online condition monitoring applications. The consistency parameter has long been used as an indicator of SK filtering effectiveness [27,28]; therefore, it was considered to further evolve this parameter in an attempt to extend its usefulness. Adapting SK technology to operating conditions allows for enhanced diagnosis effectiveness across the operating envelope of the rotating machinery and by creating a more computationally light method this will be an enabler for online condition monitoring while a gearbox (or other mechanical system) remains in action.

Novel aspects of this study:

- Propose a novel consistency vector that enables multiple consistency bands to be automatically detected, evaluated and critical values retained, with no user input
- Propose a novel consistency vector that incorporates both the SK values and the number of realizations across which the peak is consistent, thereby increasing the sensitivity and granularity of the measure
- Perform novel experimental investigation of these two consistency vectors alongside machine learning algorithms to enable prediction of optimal SK technology parameters to data collected in different operating conditions.
- Novel comparison of the proposed consistency vectors with the traditional consistency technique
- The main objectives of this study are:
- To propose and develop two novel consistency vectors allowing multiple frequency bands to be automatically identified and stored—one of the new vectors shall contain consistency percentages, the other will contain actual SK peak values
- To propose prediction of the total probability of correct diagnosis by employing the proposed two novel consistency vectors
- To experimentally validate that these two new vectors can be used to predict the total probability of correct diagnosis when applying many combinations of SK technology parameters to data
- To compare the machine learning results using each of the novel parameters as input data, and also contrast to the traditional consistency technique, in regard to prediction of diagnosis probability, demonstrating the gains of the newer methods
- To demonstrate that the novel machine learning techniques are able to optimally adapt the Spectral Kurtosis for gear fault diagnosis.

As the consistency parameters (traditional and novel) are quick and simple to compute, it is proposed that many combinations of SK technology parameters can be evaluated in a short time as an adaptation method to optomise final diagnosis. This increase in speed and reduction in processing power allows for more combinations of SK resolution and threshold to be tested, while also enabling more online use of adaptation techniques and optimisation of diagnosis effectiveness. This paper aims to improve real-world applicability of SK techniques by proposing and experimentally validating a methodology for optimising SK based diagnosis of gearboxes, run in multiple combinations of operating conditions. This is carried out using a computationally light machine learning technique, well suited to on-line implementation to adapt the Spectral Kurtosis technology to the speed and load conditions.

The paper first discusses the methodologies utilised in this research; namely the Spectral Kurtosis (Section 2.1 and Section 2.2), novel consistency vectors (Section 2.3) and their estimation, along with the employed machine learning processes (Section 2.4) and the error scores used to compare machine learning processes. An experimental setup is then described (Section 3.1) along with the signal processing techniques employing for analysing the data and undertaking the presented research. Finally, results show the effectiveness of machine learning on the data (Section 3.2, Section 3.3 and Section 3.4), the estimation time savings (Section 3.5), achievable when utilising novel consistency vectors and machine learning versus the computationally heavy traditional method, plus an example of prediction of optimally performing SK technology parameters for a dataset (Section 3.6).

## 2. Methods

### 2.1. The Spectral Kurtosis Technology

The Spectral Kurtosis is a powerful tool in the condition monitoring of gears, bearings and other rotating machinery. The Spectral Kurtosis is the application of the statistical measure of Kurtosis to the frequency domain content of a signal and is the 4th order spectral moment [29]. The calculation is a measure of “distance from Gaussianity”, or more generally, how close to a normal distribution the frequency content is—the sign of the output defines if it is more or less peaked than normal. Being a measure of peakedness, the Spectral Kurtosis is effective at identifying signal impulsiveness [30], with the technology showing a particular sensitivity to non-stationary variations in signals and can detect the frequencies containing these changes [6,7,8].

The Wiener filter is still considered one of the most superior filtering techniques in the signal processing domain, and, as discussed above, is very similar in composition to the Spectral Kurtosis, with $W\left(f\right)\;\propto \sqrt{K_{x}\left(f\right)}$, where *W(f)* is the Wiener filter, *K_x_(f)* is the Spectral Kurtosis of time domain signal *x*, and *f* is the frequency.

**Figure 1:**
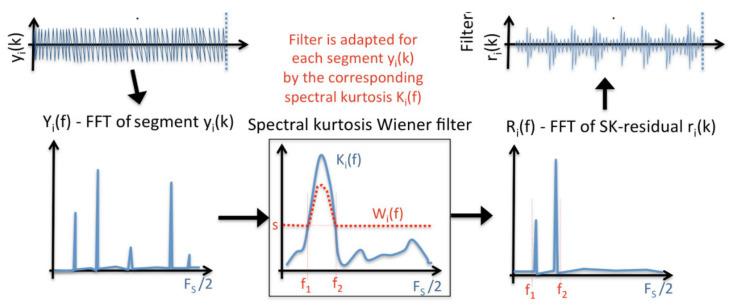
Pictorial representation of the SK derived Wiener filter with threshold ‘S’ [31].

To generate a Wiener filter, frequencies with SK values above a pre-set threshold (the SK threshold) are included in the filter, but all other frequencies are set as $W\left(f\right)\;=\;0$, effectively isolating and enhancing small non-stationary transients. This resultant filter is commonly referred to as a Spectral Kurtosis derived Wiener filter, and is now a standard tool to isolate damage related changes in a signal’s frequency content [8]. As demonstrated in Figure 1, the Wiener filter behaves as a bandpass filter in the frequency domain, which when applied back to the classical residual signal isolates and passes the signal components related to damage.

The time domain vibration signal is divided into many shorter realizations, using convergence analysis to determine the number of shaft rotation averages required to create a consistent TSA signal trace [5] without over averaging. This TSA is then converted into a classical residual signal by removing the mesh frequencies and shaft harmonics [1].

To evaluate the Spectral Kurtosis on measured vibration data, the classical residual signal undergoes several further processing steps. First, the Hanning window function is defined, with a length, specified by the SK frequency resolution. This window function is used because it has gradual increases in amplitude, touching zero at each end and, therefore provides an effective suppression of spectral leakage which can enhance FFT performance while having minimal disturbance of frequency resolution [32,33]. On non-periodic signals, as used in the Fourier transform, the Hanning window is able to improve amplitudes of interest when compared to the use of a rectangular window function [34]. The value of SK resolution can either help or hinder the separation between damaged and undamaged data by performing too much or too little smoothing during FFT calculations.

As the Matlab programming environment was used for this investigation, the spectrogram command was utilised so that in a single processing step the window function is progressively moved across a single realization of the time domain signal with a specified overlap between windows, with the Short-Time Fourier Transform (STFT) calculated for each window iteration. From the STFT, the squared envelope can be evaluated by squaring the absolute amplitudes of frequency content, which are then stored in matrix form. All of the individual squared envelope calculations are averaged in order to compute the power spectrum and from this the resultant SK:

(1) $$K_{x}\left(f\right)\;=\;\frac{S_{4,x}\left(f\right)}{S_{2,x}\left(f\right)}-2$$

where $S_{n,x}\left(f\right)\;=\;{\langle \left|X\left(t,f\right)\right|}^{n}\rangle$ and is the squared envelope of signal *x(t)*, while 〈·〉 represents the time averaging operator, ie, averaging across the various time domain windows of the *spectrogram* output matrix.

The Wiener filter is derived by evaluating where the SK is above a pre-set threshold, creating a bandpass filter which is multiplied element-wise with the SK data, before having the square root taken. This is a frequency domain filter, which when applied to the classical residual of the original data, generates the SK residual which is then used for damage diagnosis and contains mostly damage related energy.

The SK technology parameters, SK resolution (SKres) and SK threshold (SKthres) [8] must be optimised to the dataset being analysed as they have a substantial impact on the derived Wiener filter and its overall effectiveness for isolating damage. The SK resolution is directly linked to the amount of smoothing that is performed during the Fourier transform process, so can over or under average the data. Similarly, if the SK threshold is excessively high or low it will allow either too little or too much of the signal to pass through. Either of these scenarios can be detrimental to damage diagnosis as diagnostic information could be missed or the SNR could be lowered due to non-damage related noise passing through the Wiener filter.

The SK resolution is often normalised as a function of mesh period to provide more context to the selection and because an SK resolution around mesh frequency is often a good initial guess for processing [28]. In reality, the SK resolution when applied to the signal is non-dimensional, i.e., a frequency is specified, but this is then evaluated into a number of points based on the sampling frequency before being converted into an equivalent length of the Hanning window, which has a length of N points. In the absence of any prior information on the dataset, a typical SK threshold used is the 1% significance threshold, which is calculated based on the number of Hanning windows used per realization and therefore the number of frequency traces used in the averages when calculating the power spectrum and Spectral Kurtosis. The 1% statistically calculated threshold is defined as the level above which a point above this value has only 1% chance of being caused by a transient and 99% chance of being related to damage. This method produces an SK threshold that usually performs well but is not always the optimum choice.

### 2.2. Adaptation of the Spectral Kurtosis Technology to Varying Operating Conditions

The authors have previously highlighted the importance of adapting SK technology parameters when gearboxes are run in varying conditions of speed and load [20,21]. Correctly adapting the processing variables can significantly improve the diagnosis of gearboxes in varying operating conditions. Real world rotating machinery often have several sets of standard operating conditions (idling, accelerating, cruising, etc.), which is contrary to the typical laboratory research with a test rig operated at constant speed and load with the only changing variable between datasets being the presence of damage. This latter scenario makes it possible to create highly tuned diagnosis algorithms, but they may not be easily applicable to situations outside of the research environment where diagnosis needs to take place during all operational states. As such, this research focuses on diagnosing gearboxes with step-changes in operating conditions, though, the techniques and results could also be applied to other rotating machinery with other changes in operating states.

Changing operating conditions leads to different frequency bands being related to damage and, therefore, passing through the Wiener filtering process, so it is reasonable to see that the SKres and SKthres which define the Wiener filter are both heavily linked to these frequency bands and the final diagnosis probability. Many combinations of SK resolution and threshold will be applied to both damaged and undamaged data, in multiple combinations of speed and load, recording the overall probability of correct diagnosis in each case, which will be used later in the machine learning process.

### 2.3. The Traditional and Novel Consistency Vectors

Gelman [27] defined the traditional consistency parameter as a technique for measuring frequency bands with high SK peaks across many realizations The consistency parameter allowed a numerical quantification of the percentage of realizations, containing SK peaks in defined frequency ranges. Any SK peak above the threshold garnered a 1 value for that realization, or no peak resulted in a 0, and, therefore, a percentage consistency could be calculated over all the realizations. This method assumed single peaks would be at the same frequency in each realization. Peaks above the SK threshold form the derived Wiener filter, and it was therefore proposed that the higher the consistency, i.e., the more realizations having similar frequency peaks, the increased likelihood that the detected peaks are truly damage related rather than noise. The consistency parameter in [27,28] is not employed for prediction of the total probability of correct diagnosis in previous work.

Further development of the SK consistency process has facilitated the authors to develop automatic identification of multiple consistent bands across the realizations of a data set, while also recording more data for each peak. This has led to the creation of two novel consistency vectors. Equal frequency width binning is applied across the entire frequency range, with individual bins being evaluated on each realization to identify SK peaks above the threshold. If one exists, this is recorded as a 1 in the first consistency matrix, along with the actual SK value of the peak in a second consistency matrix. A second pass binning process is then performed to identify SK peaks, that are spread across multiple bins—effectively variable width binning—in which case, all included bins are set to 1 in the matrix and the peak SK value is taken.

The result is two matrix of size *m*, *n* where *m* is the number of frequency bins, and *n* is the number of realizations; one matrix contains the consistency value (0 or 1) and one the value of the SK peak (or 0 if no peak above the threshold). Once all bins in all realizations have been analysed, the consistency percentage per bin are calculated by averaging the matrix across all realizations (i.e., along the n dimension) as follows:

(2) $$p_{b}=\frac{\sum CM_{b}}{Nreal}\times 100$$

where *p* is the traditional consistency parameter per bin (in percent), *CM* is the first consistency matrix for each bin, *Nreal* represents the total number of realisations and subscript *b* denotes the bin number [20]. This is then recorded as the first novel consistency vector, the components of which are the traditional consistency parameters for multiple variable width bins.

This process is repeated for the second consistency matrix, containing SK peak values. The final two novel consistency vectors will each have a size 1, m, where m is equal to the total number of variable width frequency bins, containing consistently high SK data. The result for each automatically identified and analysed frequency bin is considered a consistent frequency band in the vibration data.

This novel process has several outcomes. Firstly, frequency bands with high SK values, that are above a pre-set threshold and are consistent across many realizations, are identified. These are the frequency bands, that are unlikely to be due to signal discrepancies or noise and, thus, in “damaged” data files are likely to be damage related. As with the traditional consistency parameter, the higher the consistency of these regions, the more realizations are displaying SK peaks in the same frequency bands, and therefore there is more similarity between the vibration signal across all the realizations. This then increases the likelihood that these frequency bands are not due to noise and must contain contained damage related impacts. It is assumed that there would not be consistent frequency bands in “undamaged” data, however, if they were to appear, then it is an indication, that the SK threshold is low.

**Figure 2:**
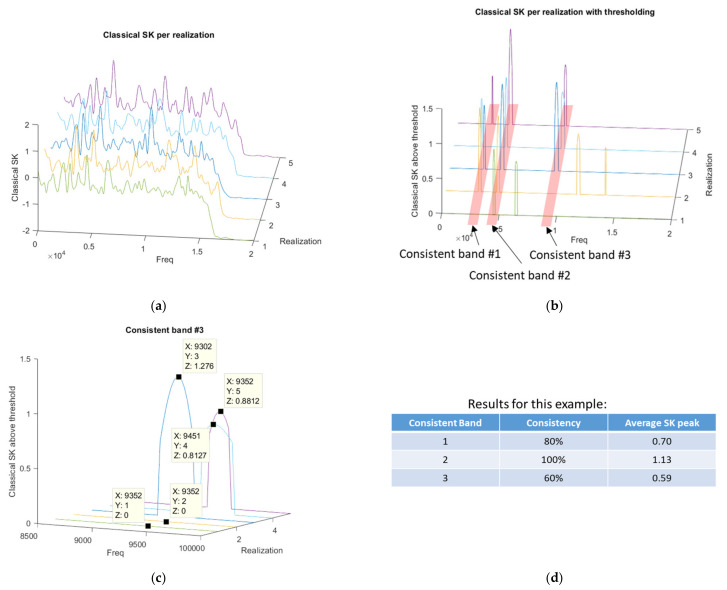
Representation of the differences between the first novel consistency vector and the second novel consistency vector with SK values, considering a 5 realization example; (**a**) Classical SK per realization; (**b**) Identification of SK above the SK threshold and resulting consistent frequency bands; (**c**) Calculating the SK peak values for each consistent band; (**d**) Comparison of the resultant novel consistency and novel consistency with SK values.

Secondly, the new process also identifies the SK data in each consistent band for further evaluation. Rather than just summarising the consistent bands as 0 or 1 values and a final percentage, the actual SK peak value is retained for each realization in the second novel vector. By incorporating the SK values into the consistency vector, it is expected that there will be more sensitivity when looking for a correlation between the second novel vector and the total probability of correct damage diagnosis. Incidentally, due to recording a 0 for SK in realizations with no peak, when averaging the SK data across all realizations, this, effectively, also considers the percentage of realizations with a peak. This process is demonstrated in Figure 2, considering a 5-realization example, whereby, after automated variable width binning, 3 consistent frequency bands would be identified and evaluated.

Lastly, and equally as importantly, the novel consistency vectors retain information from multiple frequency bands, which allows frequency bands with lower consistency to also be recorded in the matrix alongside the highly consistent frequency bands. The additional information is retained without jeopardising the traditional methods for maximising diagnosis effectiveness—the traditional technique is to only look at frequency bands with high consistency values as they are the ones related to damage. To obtain the best chance of a correct damage diagnosis, the damaged data would have highly consistent band(s), while the undamaged data ideally have no consistent bands at all. As already discussed, there can be explanations for bands in undamaged data such as low thresholds or other vibration sources.

However, on some data sets, certain combinations of SK resolution and SK threshold will result in SK peaks above the threshold beginning to appear in the “undamaged data” as well as the “damaged data”. These SK peaks in “undamaged data” are not damage related, and are typically a function of the SK threshold being too low and/or the SK resolution being sufficiently short as to result in a “spiky” classical SK. However, the SK peaks in “undamaged data” still have the ability to reduce diagnosis effectiveness as they can lead to false alarms in the diagnostic process.

For data collected for each combination of speed and load, many different sets of SK resolution and SK threshold are applied, with the novel consistency vectors recorded per combination of SK technology variable pair. As the aim of this research is to propose the two novel consistency vectors to predict total probability of correct diagnosis, the machine learning algorithms require as much data as possible to fully distinguish between the various inputs and results. By including lower consistency areas in the novel consistency vectors via lower SK thresholds, i.e., frequency bands with K peaks in “undamaged data”, the machine learning algorithm is able to better predict false alarms in the undamaged data.

### 2.4. Machine Learning for Prediction of Diagnosis Effectiveness

The main proposal of this research is to predict the final overall diagnosis effectiveness when adapting SK technology parameters. The consistency vectors contain multiple dimensions, so, in order to assess the correlation between consistency vectors and the total probabilities of correct diagnosis and predictive abilities of consistency vectors, machine learning techniques is implemented here.

Of the commonly utilised regression modelling techniques, a Gaussian exponential regression model [35] was applied to the data and performed well. Regression models are used to predict an output from various input dimensions when using supervised learning. Gaussian processes are stochastic and utilise Bayes rules of probability to describe the distributions over functions instead of scalars or variables, making predictions based on probability density functions with a mean and variance, and thereby producing confidence intervals on the predictions [36]. Gaussian processes are relatively simple yet are often able to reproduce the properties of more complex machine learning methods, e.g., neural networks [37].

**Figure 3:**
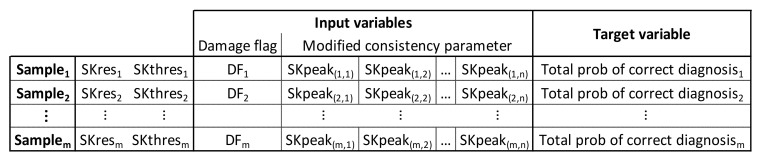
Example of machine learning variables, when using the novel consistency vector, related to SK peaks. The damage flag (DF) is a 0 or 1 value to denote if the sample was from an SKres and SKthres applied to a damaged or undamaged data file.

The machine learning process is performed separately for each combination of speed and load. The input vector is a combination of the consistency vector being investigated (either traditional, or one of the two novel) plus a damage flag (0 for undamaged gearbox, and 1 for damaged gearbox), and the target output being the total probability of correct diagnosis. Each combination of SK technology parameters is applied to both a damaged and undamaged data file. Depending on if the file is damaged or undamaged a flag will be recorded in the input variables (1 = damaged, 0 = undamaged), and once all realizations have been processed the calculated consistency vector is also added to the input variables. This represents an input sample for that speed and load case. It is therefore clear that for each combination of SK resolution and SK threshold there will be two samples in the machine learning data, corresponding to a damaged and undamaged data, respectively. An example of the variables, used for machine learning, is shown in Figure 3, demonstrating the input vector and target variable in the columns, with each row being an individual sample.

For this research, to utilise the traditional consistency parameter as the input variable a single consistent frequency band was used, hence the entire input vector per sample (as displayed in Figure 3) is the damage flag and the one consistency value. For the novel consistency vectors, up to 10 consistent frequency bands were identified, analysed and recorded, which allowed all of the data to be captured, and the consistency matrix to be a constant size. Few data files had this many consistent areas so the empty cells were filled with zeros. Even though this ensured all data were recorded, not all consistent bands were used (or required) in the machine learning process. A sensitivity study was conducted to assess the impact of using too many or too few consistent bands, as with all machine learning using an incorrect number of dimensions can be detrimental to results.

The Gaussian exponential regression model was trained using 5-fold cross validation. This means that the complete dataset is randomly split into 5 partitions, with 4 used to train the model and the final portion (the holdout set) to test the model. This process is repeated 4 more times to use all of the train/test data combinations. As such, each data point will be used in the hold out set to test the model once, and in training sets 4 times. In this instance, the dataset had over 1300 samples due to the number of combinations of SK resolution and threshold being evaluated, i.e., more than 30 SK resolution options, over 30 different SK thresholds and all of these for both “damaged” data and “undamaged” data. As described in Figure 3, for the novel parameters there were a maximum of 11 input dimensions (a damage flag plus up to 10 consistent frequency bands) plus one target, therefore, the maximum machine learning dataset size is over 1300 × 12.

**Figure 4:**
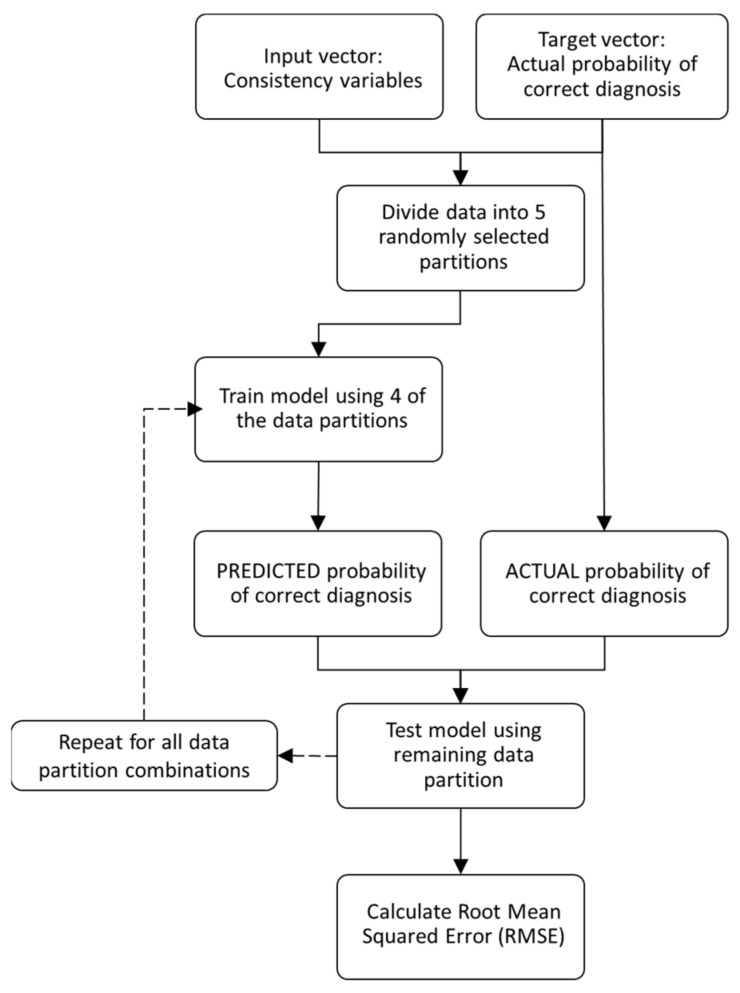
Flowchart of the machine learning process.

The final step is to calculate an error measure to evaluate the performance of the trained model. In this research, the mean absolute percentage error (MAPE) was selected as this metric is most relatable to the initial dataset and, as expressed by Moreno et al. [38], has many useful features including unit-free measure, ease of interpretation and clarity of presentation. The MAPE calculation does have limitations, particularly, when actual target values are small or close to zero [38]. A small absolute error can lead to a large percentage error, so these situations must be handled carefully. The most commonly proposed method is to set upper and lower bounds of data, so, as not to divide by excessively large or small values [39]. This limitation is particularly relevant in this research as the range of SK resolution and SK threshold, used for machine learning, are specifically wide and varied, so, some combinations are expected to have low diagnosis effectiveness. For this reason, the models were trained, but the MAPE was evaluated only for ‘useful’ data, with a lower threshold as 75% of the total probability of correct diagnosis and upper set at 100% of the total probability of correct diagnosis. In practical application, SK technology parameter pairings giving a total probability of correct diagnosis below the lower limit would not be used for gearbox condition monitoring and be discarded anyway. A flowchart representing the machine learning process is shown in Figure 4.

The MAPE is a measure of the percentage error between actual total probability of correct diagnosis and that predicted by the model. The percentage error is calculated for every sample point then averaged to produce the MAPE value, which shall be used to evaluate how well the model can use the input variables to predict the target.

## 3. Results

### 3.1. Test Rig and Data Processing

**Figure 5:**
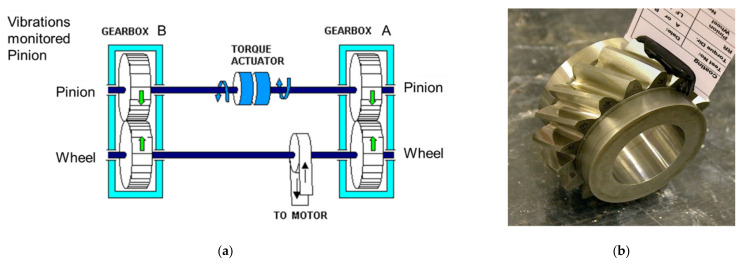
(**a**) Representation of the back-to-back test setup; (**b**) 16 tooth pinion when new (teeth labelled 1–16).

Data were acquired from a test rig, comprising a pair of identical back-to-back gearboxes having 16 teeth on the pinions and 24 teeth on the gears, i.e., a ratio of 1:1.5. These were linked by two torsionally compliant shafts, connected to a motor and servo-hydraulic torque actuator, respectively (Figure 5a). The test rig allows precise control of both speed and load over a wide range of operating conditions, whilst also allowing different variations of helical gear to be tested. During this test, the gears had a helix angle of 30°, a face width of 25 mm and were manufactured from S156 steel (Figure 5b). Due to the design of the meshing gears, vibration signals will never be a result of a single pair of meshing gears, therefore, when evaluating damage per tooth there will be some merging between the diagnosis of successive teeth. However, in this case the damage was distributed throughout the gears meaning this was not an issue. The test apparatus was located in the Gear Research Centre at Newcastle University, with data collected by L. Gelman, K. Gryllias, and M. Vaidhianathasamy.

Two sets of operating parameters are investigated for this research: speed of 1500 rpm and torques of 250 Nm and 500 Nm The experiment design included three main stages: (i) capturing of experimental data of undamaged gearbox for variable load conditions; (ii) creation of fully controlled experiment conditions, under which development of natural pitting occurred in multiple gearbox teeth; (iii) capturing of experimental data of damaged gearbox for variable load conditions. Firstly, experimental data were collected for both combinations of operational conditions with the gearbox in an undamaged state. Next, the gearbox was run in a closely controlled set of operating conditions (high speed and high torque), under which natural occurring putting damage occurred in multiple teeth. Each 10M cycles of the pinion, the gearbox was disassembled and all tooth surfaces were subject to evaluation of pitting severity. Once stereo optical pitting evaluation was completed, the pinion and gear were installed back into the gearbox in the same angular orientation as before and the test was continued. The final experimental step was to gather data, related to the damaged gear state, in each combination of operating conditions.

Early stage gear tooth faults are known to present challenges for reliable detection [40], with pitting being a typical stage of a fatigue related damage likely to occur on industrial gearboxes. Pitting is a mechanism of material loss and takes place on the contact surfaces; in this case, on the flanks of the interfacing teeth. With even small amounts of material loss, the surface profile and mesh contact stress of individual teeth varies, leading to a self-propagating damage and continued destruction. This process inevitably increases sound and vibration from meshing contacts and also due to any resultant gear imbalance, leading to sub-surface micro-cracks and spalling, which can eventually lead to tooth fracture and loss [41,42]. As such, early pitting detection can prevent additional continued gear damage via gear maintenance action and is vital across many applications, e.g., renewable energy, wastewater treatment, aerospace, etc.

**Figure 6:**
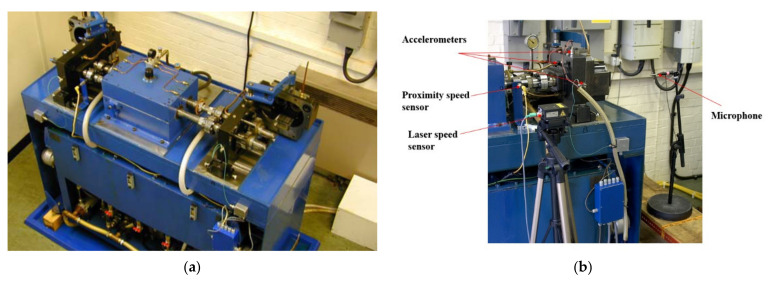
(**a**) Overview of experimental gearbox setup; (**b**) Detailed sensor locations.

LabView was used to record data at 40 kHz sampling rate, with vibration and two speed sensors—proximity and laser (Figure 6). The positions of the various sensors are demonstrated in Figure 6b. The accelerometer was surface mounted on the gearbox casing as close as possible to the meshing gears to reduce any effects on the vibration signal, caused by the transmission path. The accelerometer was rigidly attached, using the threaded holding the base to prevent any relative motion between the sensor and gearbox housing.

The accelerometers used to record vibration data during gearbox operation were KCF AG107M linear sensors, detecting vibration through shear mode, positive polarity, piezoelectric transducers. The full sensor specifications can be described as: Featuring sensitivity of 50 pC/g, transverse sensitivity below 5%, flat frequency response in the frequency range (0.5–6000) Hz (±1 dB), 80 g maximum measured acceleration and mass of 28 g. Piezo material of the accelerometer was PZT-5, isolation resistance was more than 10 × 109 Ohms, capacitance was 1200 pF, temperature range was −40 to +150 deg C, shock limit was 800 g, temperature sensitivity is 4 mg/deg C, structural strain sensitivity was 0.2 mg/micro strain and magnetic field sensitivity was 2 g/T. The output connector featured water proof sealing [43].

**Figure 7:**
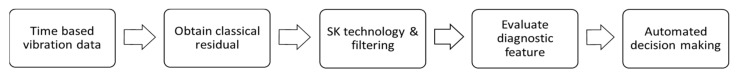
Flowchart of diagnosis methodology.

The signal processing techniques have been explored in depth throughout Section 2, and follow a typical processing flow for vibration data collected from gearboxes (Figure 7).

**Figure 8:**
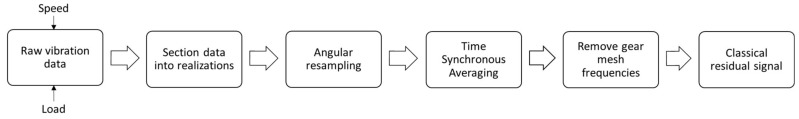
Flowchart of obtaining the classical residual signal.

A tacho pulse, derived from the speed sensors was used to enable time synchronous averaging which has the effect of removing non-stationary signal components. Using a realization length of 8 s ensured convergence of the TSA in all speed and load conditions. Mesh frequencies were then removed using a comb filter to leave the classical residual (Figure 8).

**Figure 9:**
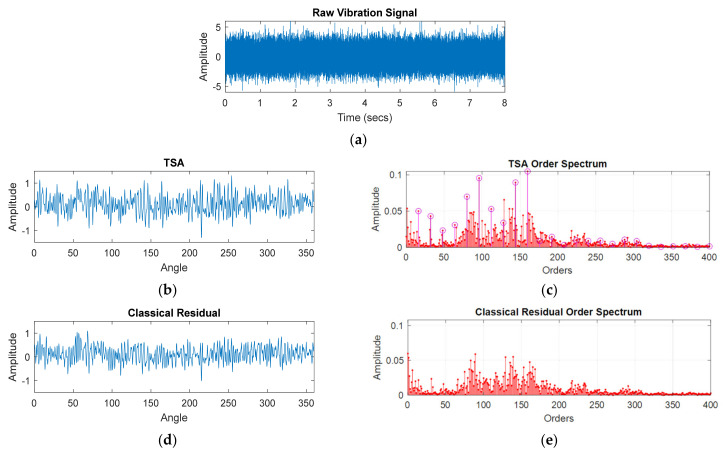
Signal processing flow demonstrated with real data; (**a**) Raw time-based vibration data; (**b**) TSA signal represented as a one complete rotation of the gear; (**c**) Order spectrum of the TSA signal, with gear mesh frequencies clearly visible in pink; (**d**) Classical residual signal; (**e**) Order spectrum of classical residual shows dominant mesh frequencies removed.

The effect each processing step has on the vibration signal is demonstrated in Figure 9.

**Figure 10:**
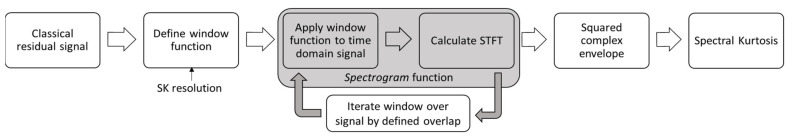
Flowchart of evaluating the Spectral Kurtosis from the classical residual (see Section 2 for further detail).

**Figure 11:**
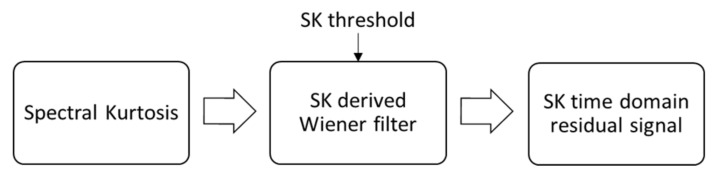
Flowchart: from the Spectral Kurtosis to the SK residual.

For each set of SK technology parameters, the SK was calculated (Figure 10), and the Wiener filter derived (Figure 11), during which the consistent frequency bands could be identified and both traditional and novel consistency vectors evaluated (full detail in Section 2.3).

Applying the Wiener filter to the classical residual signal gives the SK residual signal (Figure 11), after which the Hilbert transform was used to obtain the analytical signal and to calculate the SK residual squared energy envelope, which is used for a final damage diagnosis.

Using the Hilbert transform to compute an analytic signal is comparable to amplitude demodulation in envelope analysis. There are several advantages to using the envelope during SK techniques, most notably, that analysis can be performed on distinct frequency bands, which limits interference from other similar frequency components [44]. Ho and Randall concluded, that using the envelope spectrum (or squared envelope spectrum if the SNR is greater than 1) means that defect frequencies are more visible [45].

Automated decision making is implemented using cluster analysis and k nearest neighbours methods [9]. The initial centroids are randomly seeded, and clusters are formed by assigning each diagnostic feature point to a centroid, based on smallest Euclidean distance. Once all feature points have been assigned, the centroids are re-calculated as the mean of all the points currently assigned to that cluster. The cluster assignment process is repeated until no feature points change cluster between iterations and the centroids are therefore fixed. Within each cluster, the distances between each point and all others are calculated, with the minimum k nearest neighbour distances averaged and recorded. During training, the maximum nearest neighbour value is the measure of cluster size; so, when the process is repeated with test data, if the minimum neighbour distance is larger than the training value. Then a feature point is considered to be outside of a cluster and a single anomaly detection is flagged.

**Figure 12:**
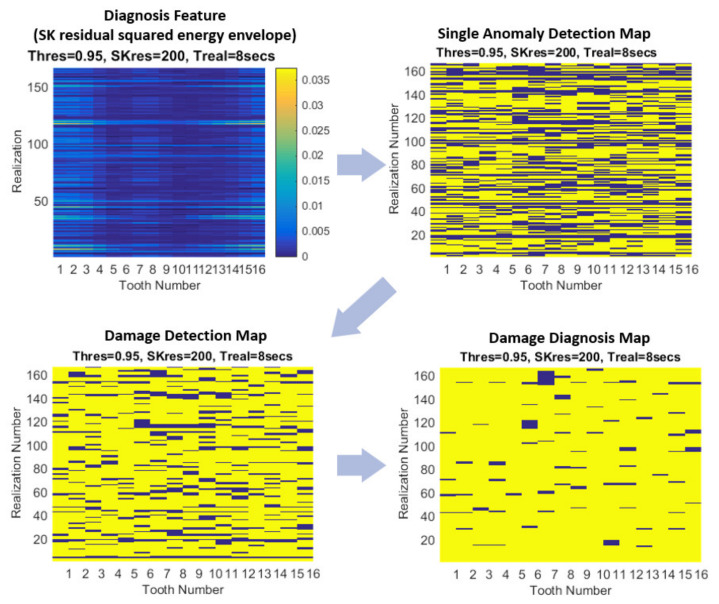
Example of automated damage diagnosis process.

A maximum of 6 clusters is used, along with 5 neighbours and these values are kept constant throughout the study. Results are compared to a training data set (also kept constant throughout the study), from which single anomalies are detected. These single detections then have weighted majority rules applied to form a damage detection map, before grouping and application of further weighted majority rules to give a damage diagnosis map. An example is demonstrated in Figure 12, whereby the diagnosis feature is shown on the top left, followed by the single anomaly detections and then the weighted rules are applied to give damage diagnosis maps.

As the damage was evenly distributed across all teeth, total probability of correct diagnosis is evaluated by the percentage of area of the damage diagnosis map that shows the correct diagnosis, i.e., in undamaged case there should be no damage highlighted (all blue), and in an ideal damaged case there should be 100% highlighted (all yellow). This calculation is used because the pitting damage was naturally seeded and evenly distributed across all teeth.

**Figure 13:**
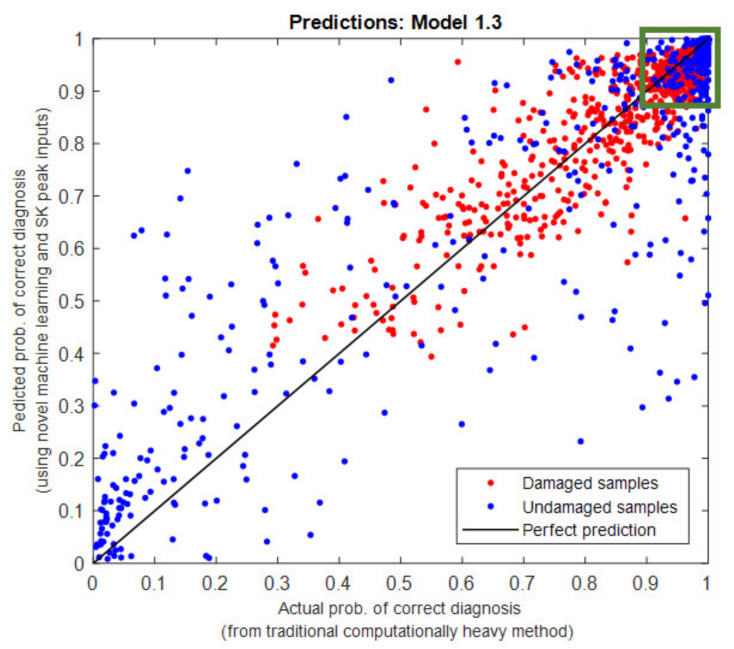
Machine learning output visualisation.

Applying the machine learning algorithms (described in Section 2.4) allows a model to be trained and tested that predicts the probability of correct diagnosis from the consistency vectors. An example of the output from the machine learning process can be seen in Figure 13, whereby each dot represents a sample point in the dataset. The best performing combinations of SK technology parameters are highlighted in green, and a perfect predictive model would have results along the black line. For each combination of SK technology parameters, the damaged and undamaged results then need to be combined to obtain an overall effectiveness of diagnosis.

An equivalent output is generated for all combinations of the machine learning process undertaken in this research, e.g., for different operating conditions, different consistency input variable and varying number of dimensions (number of consistent frequency bands to use in training the model).

### 3.2. Sensitivity Analysis on Number of Consistent Frequency Bands to Use for Machine Learning Input

As discussed in Section 2.4, during evaluation of the novel consistency vectors, it was allowed for up to 10 consistent bands to be included in the result vectors. However, these may not all be helpful for diagnosis; so, when generating the input vectors for machine learning, the number of bands can be limited to use only a certain number, i.e., restricting *n* in Figure 3. A sensitivity analysis was performed for each speed and load to evaluate the effects of different length input vectors, with results represented using the RMSE values of the machine learning process, which are shown in Table 1. The bands were always selected in descending order, with band 1 being the most consistent and band 10 the least consistent. This approach was chosen as more consistent bands passing above the SK threshold and used for diagnosis are most likely to be damage related rather than random noise or other signal discrepancies.

Regardless of operating conditions, best results are typically found when using the 3–6 most prominent consistent frequency bands for the analysis. This number varies due to the spikiness of the classical SK varying and, therefore, different speeds and loads exciting unique damage related vibration frequencies. Whilst each speed and load combination has a different optimum number of bands, the difference in MAPE between the results within the 3–6 band range is small and the models will all perform well.

Simply using one frequency band does not perform well because these data have more than one frequency band attributed to damage, meaning that restricting the machine learning input to just one frequency band excludes too much of the damage related frequency components for the trained model to accurately predict the total probability of correct diagnosis. Using one band is the comparable to using the traditional consistency parameter for a machine learning input. Conversely, when using too many consistent bands, the machine learning process has excess information in the input vectors that is not related to damage. There are two possibilities for this outcome, the first is that many of the samples (SK resolution/SK threshold combinations) do not have this many consistent bands which means the higher dimensions are populated by zeros in the consistency vectors. Having many zeros in the inputs mean that the machine learning cannot use that dimension to distinguish between samples. This is typical of an undamaged data file that will have very few entries in the consistency vectors, and even these will have low values.

Alternatively, in damaged data sets, the diagnostic information is concentrated in certain number of frequency bands, and the higher dimensions above this typically contain noise or SK peaks, not related to damage, that have passed through the SK filtering process.

### 3.3. Proof of Gains and Comparison of Techniques

**Figure 14:**
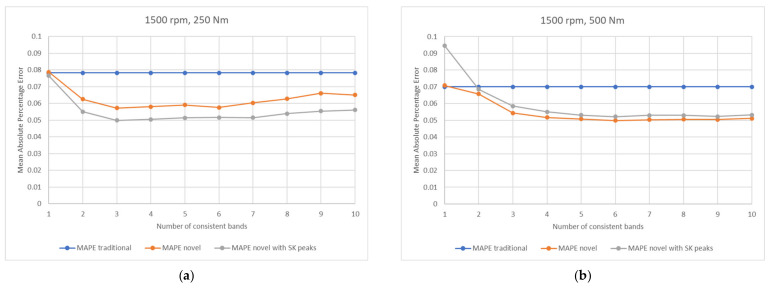
Results comparing operating conditions, techniques, and number of dimensions to use during machine learning. All charts have been scaled identically to aid in comparisons; (**a**) low torque results; (**b**) high torque results.

For each set of operating conditions, the machine learning process was completed using each of the input variable options—traditional consistency, novel consistency and novel consistency with SK values—along with varying the number of dimensions in the input variable, i.e., how many consistent frequency bands to use. The results for each of the set of gearbox operating conditions are displayed in Figure 14.

Multiple conclusions can be drawn from the charts. Both novel parameters outperform the traditional consistency when training the machine learning models. This is due to the excitation caused by damage being distributed across several frequency bands, meaning multiple bands are required for accurate diagnosis, a feat which is not achieved using the traditional consistency.

In the low load (250 Nm) data, the MAPE values show that the second novel vector outperforms the first novel vector regardless of number of bands used; however, in the higher load data (500 Nm) there is little to no separation between the novel parameters, with both performing comparatively well. While the separation is small, in 500 Nm data, the MAPE novel narrowly outperforms the MAPE novel with SK peak data. The difference in performance is minor enough that it is not statistically significant (statistical significance of the findings shall be discussed in more depth in Section 3.4); so, the results are comparable.

The addition of SK peak data enhances sensitivity to the machine learning model by increasing the likelihood of being able to better distinguish between consistent frequency bands, i.e., if two bands have the same consistency percentages (i.e., components of novel consistency vector), they are unlikely to also have the same SK peak heights (i.e., components of novel consistency vector with SK peaks). Moreover, the addition of SK peak data can be considered as a scaling factor to the percentage-based novel consistency vector; so, if SK values are greater than 1, then the separation between individual frequency bands for damaged and undamaged gearbox conditions via novel consistency vector with SK peaks increases as well as becoming more unique.

In 250 Nm data, the SK peak values are all greater than 1, for damaged gear data, which increases the separation between frequency bands for damaged and undamaged gearbox conditions for the novel consistency vector with SK peaks; whereas, in 500 Nm data, the SK peak values are all just below 1 for damaged gear data, which is not increasing the separation between frequency bands for damaged and undamaged gearbox conditions for novel consistency vector with SK peaks. The above facts explain why “MAPE novel” and “MAPE novel with SK peaks” methods demonstrate different behaviours for 250 Nm and 500 Nm data. It is clear, that with increasing gear damage severity, the SK peak values will increase in all cases and, therefore, the MAPE novel with SK peaks is likely to improve in performance compared to the MAPE novel.

In most circumstances, as the number of consistent bands becomes excessively large, the novel techniques performance diminishes and the separation between MAPE values form machine learning using novel and traditional input vectors becomes less apparent. As described in Section 3.2, too many bands can be detrimental to the newer techniques as it simply adds irrelevant information and additional dimensions to the machine learning process.

**Figure 15:**
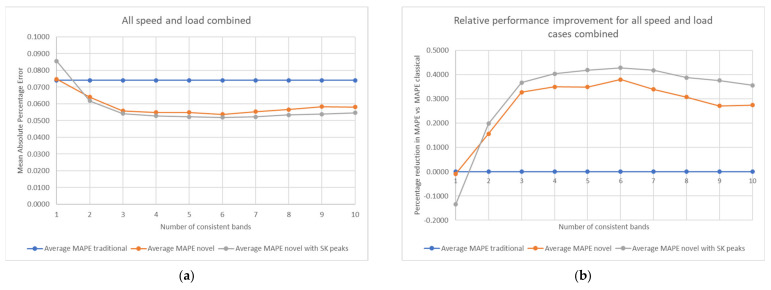
Performance of the various techniques; (**a**) absolute values when combining speed and load; (**b**) relative performance improvement with speed and load combined.

In order to generalise the techniques and allow them to be more suited to diagnosis, it is preferable to consider the number of bands used across all speed and load cases, i.e., to assess if there is a number of bands that works well in all cases. By averaging the results in Figure 14 across the operating conditions, the outcomes displayed in Figure 15 are observed.

Once the results are averaged across all the combinations of operating conditions, it is clear to see that when using consistency values to predict the total probability of correct diagnosis, the new techniques utilising the novel consistency perform better than just using the traditional consistency. The novel consistency vector incorporating SK values also shows less sensitivity to the number of consistent bands used in diagnosis.

### 3.4. Statistical Significance of Results

Tests for statistical significance were performed on the results presented and can be further explained by referencing back to the charts in Figure 14. As MAPE is used as the model accuracy metric and is a mean value, the most applicable statistical test is the one sided two sample *t*-test to evaluate if two means are statistically different [46]. This test considers the mean and standard deviation of the absolute percentage error distributions before the averaging takes place to calculate if the overall sample means (i.e., the MAPE) are actually different or could be within the realms of random chance, i.e., if the distributions overlap considerably. In this case, the tests will be to evaluate if the MAPE values using novel consistency inputs are statistically proven to be lower than when using the traditional consistency input. It is also possible to then evaluate if the MAPE value using SK peak data are statistically lower than using the novel consistency. All tests are conducted using 5% significance.

For the low load data (250 Nm), all results are statistically significant. Both novel consistency options perform better than the traditional method, and the MAPE with SK peaks is also statistically better than when just using the novel consistency. Next, considering the high load data (500 Nm) the tests show that the two novel parameter options are statistically proven to be better than when using the traditional input. However, there is no statistical difference between each of the novel consistency inputs.

**Figure 16:**
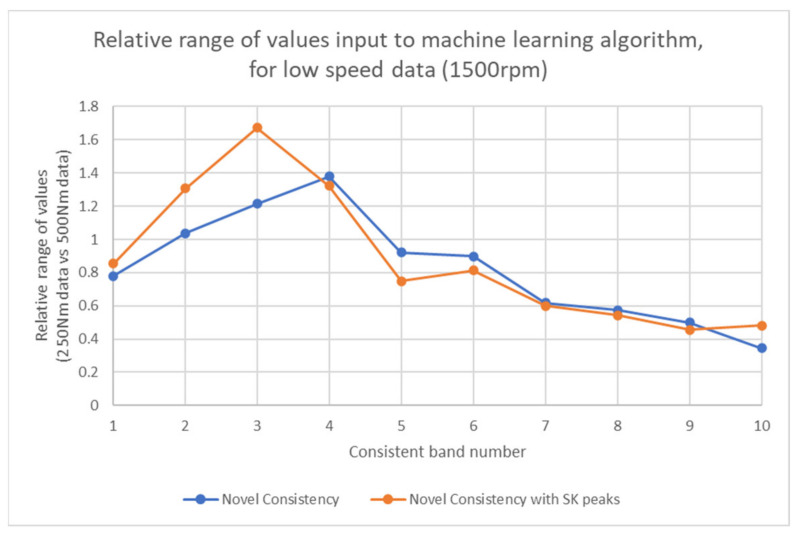
Relative range of input dimensions.

When performing machine learning, model effectiveness is improved by having greater range in the training data dimensions. In a given dimension of training data (e.g., one frequency band in this case) if there is little separation between samples then this dimension is ineffective to train the model. Conversely, if there is a large range within that dimension then it is more accurate for the model to use that dimension to evaluate the varying target variables. This explains why there is statistical significance at 250 Nm but not 500 Nm when rotating at 1500 rpm. Figure 16 shows the relative ranges between the two load cases for the novel consistency vectors. For each consistent band the chart displays the relative range of values between the data sets. This is calculated as the range of values in 250 Nm data divided by range in 500 Nm data.

It is clearly visible that in the first 3 bands, which are the ones with highest consistency and most likely to be damage related, the relative range of the SK peaks input data are much higher. This means there is a greater distribution of values in 250 Nm data than 500 Nm data, and explains why the SK peak data performs substantially better than when using the novel consistency data as an input. This proves the initial proposal that by utilising SK peak data there is additional sensitivity compared to just consistency percentages, and this is especially relevant when the SK data are close to the threshold value.

It may be possible to improve the statistical significance by having more input samples to the machine learning, i.e., using more combinations of SK resolution and threshold in the model data. More samples in the input data often improve model accuracy, and can be beneficial to statistical significance as the resultant predictions will be more precise with a smaller standard deviation.

### 3.5. Savings in Computation Time

**Figure 17:**
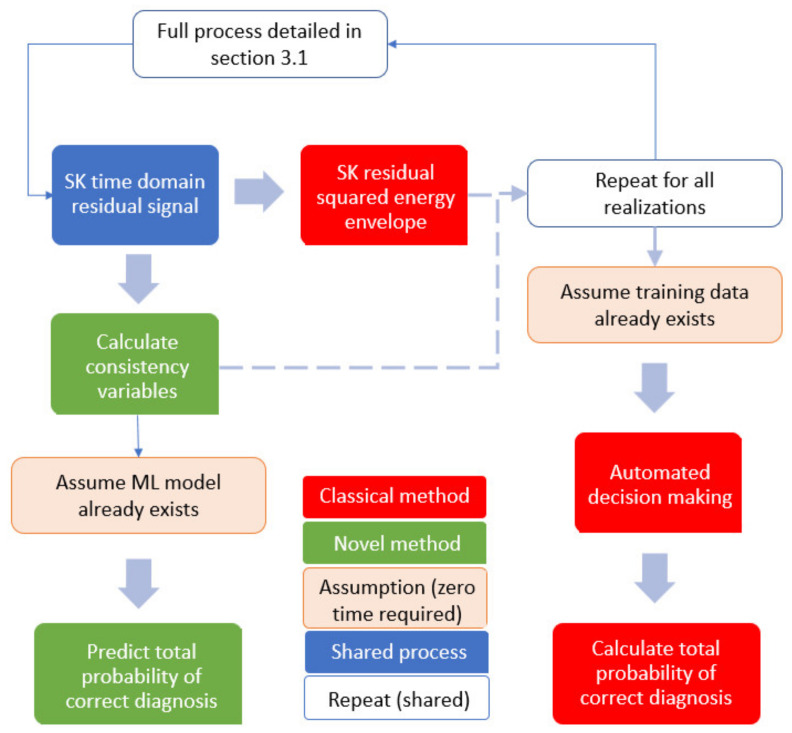
Flowchart to visualise the different estimation steps between the traditional and the novel methodologies.

The classical signal processing and diagnostic technique can be compared with the novel approach proposed in this paper to evaluate the actual computational time savings achieved by using the consistency vectors for prediction of diagnosis effectiveness and therefore adaptation of the SK technology. To make this comparison we must evaluate the differences and similarities in the computation process, as detailed in Figure 17 below. The steps highlighted in red are those exclusive to the novel calculation whereas those in green are used for the novel method using machine learning. Any blue steps are shared between both processes so the time saving estimations will be calculated twice, once including these steps and a second time omitting them. This gives an accurate representation of time saving on the specific steps this novel technique targets, but also the effect it has on the entire SK part of the signal processing flow.

When undertaking the time comparison, it is assumed that background computer processes are the same in each analysis, code is written to the same optimised standard. The analysis was performed for 9 combinations of SK technology parameters, applied to both damaged and undamaged data, which are then averaged in order to remove any bias of specific SK technology parameter values. As a result of these assumptions, the calculation times detailed below shall be used as relative measure. Running the same code on a different machine or with different background processes, etc., will change the absolute speed of calculations, but it is expected that the relative speeds remain consistent. The comparative computation times for the two methods is evaluated as follows:

As shown in Table 2, the novel method, based on consistency vectors and machine learning for prediction of total probability of correct diagnosis, has a relative time saving of up to 94% over the traditional method. Even if considering the whole SK process in the calculation the savings still amount to 81%.

Whilst the individual computation time per SK technology pair is not high—in the order of seconds—it must be remembered that in all reality many combinations will be tested to find an optimum set of SK technology parameters. The authors were consistently performing over 650 different combinations of SK technology parameters for both damaged and undamaged datasets as this covered the complete useful ranges with good granularity. Therefore, to perform all these iterations on both a damaged and undamaged data file results in over 1300 individual processing stages. If we include all common SK steps in the methods, this amounts to approximately 54 min of processing using the traditional method compared to 10 min for the novel method.

This immediately illustrates the increased applicability of the new technology to online condition monitoring as speed and computational complexity are of paramount importance. Additionally, if this technology is used offline or in a less time sensitive environment then the larger the number of combinations of technology parameters that can be tested the more likely of finding an optimum value. Quicker processing is an enabler to evaluating more SK variable combinations.

### 3.6. Adaptation of the SK Technology via the Novel Techniques

The final stage in this research process is to use the machine learning outputs and novel consistency-based methods to determine sets of SK technology parameters that will work well on both damaged and undamaged data sets. These combinations of variables can then be used to calculate final probabilities of correct diagnosis and, thereby, quickly adapting the SK technique to the operating conditions.

The results already presented in this chapter treat the damaged and undamaged data as individual samples and apply the SK threshold and SK resolution pairs to each data separately. This is why during the machine learning process there is a damage flag included as an input variable so that the algorithms know if they are expecting a damaged or undamaged result. In reality, there will be combinations, that work well on only one set of data, e.g., a high threshold will prevent false alarms in undamaged data, but may also remove diagnostic information in the damaged datafile. Therefore, the predicted probability of correct diagnosis for both damaged and undamaged data when utilising a particular SK threshold and resolution must be combined to obtain an overall measure of effectiveness of diagnosis. The combinations, that are predicted the best total probabilities of correct diagnosis, can then be compared to the combinations, that are providing the best total probabilities of correct diagnosis by direct estimation of these probabilities.

The outcomes of this comparison shall be visualised by studying the top 10 results (Table 3) when ranked by either predicted or estimated probability of correct diagnosis. This method has been chosen as the difference in diagnosis is very small amongst the best performing combinations, so evaluating the top 10 options gives a more complete representation rather than assessing just considering the top result.

The data in Table 3 was created using 3 consistent frequency bands and utilising the SK peak data as the sensitivity analysis showed this to perform well for both novel vectors. For each combination of SK technology parameters, Table 3 (2) and (3) display the actual total probability of correct diagnosis when a specific SK resolution and a threshold are applied to the dataset, alongside the total probability of correct diagnosis, what was predicted, using the proposed machine learning model. The top SK parameter combinations in Table 3 (2) and (3) are chosen, using the machine learning predictions; but, when these top SK parameter combinations are applied to the data, it would still be the ‘actual’ total diagnosis probability that results.

As an example, the topmost row in Table 3 (3) shows, that the SK technology parameters of resolution equal to 510 Hz and the threshold equal to 0.7, would result in the actual total probability of correct diagnosis of 99%. The machine learning model correctly identifies this as the best performing parameter set, though, for reference, the model predicted total probability of correct diagnosis is 97%. If the main SK technology parameters are applied to the data, you would always obtain the diagnosis probability deemed as actual total probability of correct diagnosis. Interestingly, the model created using the novel consistency vector of percentages over-predicts the diagnosis effectiveness, while conversely, the model utilising the novel consistency with SK peak data slightly under-predicts the probabilities of correct diagnosis.

The top performing combination of SK resolution and threshold calculated by the traditional computationally heavy method (Table 3 (1), highlighted green) was also identified using the novel computationally light method (Table 3 (3)). This combination of SK technology parameters is also identified in the top 10 of Table 3 (2) when using the novel consistency vectors, but it is not identified as one of the best performing parameters. In both machine learning cases many of the other top 10 results also match closely between all of the techniques. As shown in Section 3.5, this is also achieved with a time saving of up to 94%.

For the dataset analysed, using the method currently favoured in literature of setting SK resolution equal to mesh frequency and the SK threshold calculated using a 1% statistical significance [8], this would result in the total probability of correct diagnosis of 92%, which serves as a benchmark for the novel techniques. Utilising the novel SK based consistency vector and machine learning techniques identified the same set of SK technology parameters for best diagnosis as using traditional heavy methods, with a resultant SK resolution equal to 510Hz and SK threshold set at 0.7. This combination results in a total probability of correct diagnosis of 99%. For reference, these parameters are equivalent to 1.275× mesh frequency and a 0.874% significance threshold. Applying the novel techniques would therefore reduce incorrect diagnosis rate by 88% comparatively. The case using the percentage based novel consistency vector does not identify the top performing combination of SK technology parameters, but does still identify combinations that outperform the mesh frequency and 1% significance combination.

This is a significant result for increasing the relevance and applicability of the novel SK technology adaptation to online condition monitoring applications.

## 4. Conclusions

This paper proposes two new consistency vectors—the novel percentage-based consistency and the novel consistency incorporating SK peak values—for adaptation of SK based vibration diagnosis of multistage gearboxes to variable load conditions. These can be used alongside machine learning algorithms to give an early prediction of total probability of correct diagnosis. Data were collected from a pair of back-to-back gearboxes run in multiple combinations of speed and load which underwent time synchronous averaging and removal of mesh harmonics to leave a classical residual signal. SK calculations are the performed using the STFT before Wiener filters are derived and the consistency vectors calculated.

The first proposal is the novel consistency vector that automatically computes consistency across multiple frequency bands, and the second is the novel consistency vector that also incorporates SK peak values of consistent bands. The latter of these was proposed to allow additional sensitivity to the machine learning process. The signal processing has been performed using hundreds of unique combinations of SK technology parameters, with the consistency vectors recorded for each. Automated decision-making algorithms using k nearest neighbours and cluster analysis also computes a total probability of correct diagnosis for each processing iteration.

Exponential Gaussian regression was used with each of the consistency vectors in turn as inputs, and total probability of correct diagnosis as the target variable. It has been shown that the number of input dimensions, i.e., how many consistent frequency bands to consider in the machine learning, has a significant effect on the effectiveness of the machine learning model. Up to ten dimensions were analysed, with best results typically seen using 3–5 input dimensions, with the optimal number being dependent on the specific data and how damage related energy was distributed amongst the consistent frequency bands. Models were generated and tested using 5-fold cross validation, and the effectiveness was measured numerically using the mean absolute percentage error. Overall, the new consistency vectors lead to more effective machine learning models and utilising the SK data also leads to more accurate predictions when compared to other machine learning inputs.

Utilising the novel consistency incorporating SK peak values has been proven to add sensitivity to the machine learning process and improve diagnosis prediction, particularly when the SK values are close to the threshold, i.e., in scenarios with low levels of damage related energy. SK technology parameters require adaptation [1,21], when gearboxes are operated in varying speed and load conditions, though this can be a computationally heavy process. Consistency vector calculation is computationally light and quick to perform, therefore allowing quick pre-selection of SK technology parameters to a speed and load scenario.

The traditional method identified an SK resolution of 510 Hz and a threshold of 0.7 as the optimal technology variables for the dataset being used, a result that was mirrored when using the novel machine learning algorithms and SK peak consistency vectors as inputs. When analysing the top 10 combinations of SK technology parameters identified in each case they are very similar and would lead to good damage prediction and gearbox state diagnosis—in both cases better than the 1% significance and mesh frequency option. The machine learning models typically under-predicted the diagnosis probability slightly, however the trends and top performers were the same. Increasing the number of samples may lead to further increases in accuracy and reduce the prediction error.

Time savings with the new technique can be up to 94% compared to adaptation using the traditional method, with a diagnosis prediction equal to the optimal SK technology parameter pairings evaluated using the long and arduous traditional technique. The novel technique also performs better than the previously accepted best result using an SK resolution of fmesh and 1% significance threshold, with an 88% reduction in incorrect diagnosis. This makes the adaptation of SK technology more applicable to online condition monitoring and increases the relevance to industry.

## Figures and Tables

**Table 1:** Sensitivity analysis on length of input vector, i.e., how many consistent frequency bands to use; (**1**) Using novel consistency vector (**2**) Using novel consistency vector incorporating SK peak values. The colours represent the relative performance of each result, on a sliding scale from dark green (best outcome), through light green, yellow and orange, all the way to dark red (worst outcome) for each combination of operating conditions.

(1)	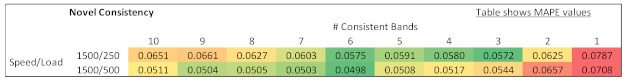
(2)	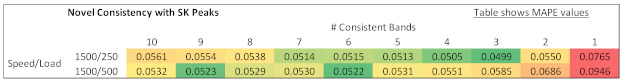

**Table 2:** Comparative computation time (seconds) between methods.

	Time taken per calculation step (second) (color coding to match Figure 17)					Totals	
Technique	SK evaluation and Wiener filtering	SK residual squared energy envelope	Automated damage diagnosis	Consistency parameter evaluation	Run all combinations through ML model	Including common steps	Excluding common steps
Classical	0.350	0.160	1.993			2.504	2.154
Novel	0.350			0.090	0.033	0.473	0.123
			Relative reduction using novel method			81%	94%

**Table 3:** Results for the top 10 combinations either (**1**) actual, (**2**) predicted using novel consistency vectors, or (**3**) predicted using novel consistency vectors with SK peak information. Both novel methods have used 3 consistent SK peaks data as input to the machine learning as this performed universally well in the sensitivity analysis. The rows highlighted in green indicate the actual best performing combination of SK technology parameters for the data and how they are predicted with the novel techniques.

			**Novel Consistency Parameter**				**Novel Consistency Parametar with SK Peaks**			
**ACTUAL top 10 combinations**			**Model PREDICTED top 10 combinations**				**Model PREDICTED top 10 combinations**			
**SK parameter**		**Diagnosis** **effectiveness**	**SK parameter**		**Diagnosis** **effectiveness**		**SK parameter**		**Diagnosis** **effectiveness**	
**Resolution Threshold**			**Resolution Threshold**		**Actual**	**Predicted**	**Resolution Threshold**		**Actual**	**Predicted**
**510**	**0.7**	**0.99**	580	0.6	0.95	0.98	**510**	**0.7**	0.99	**0.97**
550	0.65	0.99	500	0.725	0.97	0.98	520	0.7	0.97	0.97
510	0.675	0.98	510	0.6	0.96	0.98	510	0.725	0.98	0.96
560	0.625	0.98	500	0.675	0.96	0.98	510	0.625	0.92	0.96
530	0.675	0.98	520	0.675	0.97	0.98	540	0.675	0.96	0.96
530	0.7	0.98	520	0.725	0.95	0.98	530	0.65	0.91	0.96
500	0.625	0.98	**510**	**0.7**	0.99	**0.97**	510	0.6	0.96	0.96
530	0.625	0.98	520	0.7	0.97	0.97	540	0.625	0.88	0.96
520	0.65	0.98	540	0.7	0.95	0.97	480	0.775	0.97	0.96
510	0.725	0.98	530	0.65	0.91	0.97	510	0.675	0.98	0.96
(1)			(2)				(3)			

## Data Availability

The data presented in this study are available on request from the corresponding author. The data are not publicly available due to ownership and privacy issues.
